# Comparison of Manual and Rotary Instrumentation on Postoperative Pain in Teeth with Asymptomatic Irreversible Pulpitis: A Randomized Clinical Trial

**DOI:** 10.22037/iej.2016.4

**Published:** 2016

**Authors:** Bita Talebzadeh, Saeed Nezafati, Saeed Rahimi, Shahriar Shahi, Mehrdad Lotfi, Negin Ghasemi

**Affiliations:** a * Endodontist, Department of Endodontics, Dental School, Tabriz University of Medical Sciences, Tabriz, Iran; *; b *Dental and Periodontal Research Center, Department of Oral and Maxillofacial Surgery, Dental School, Tabriz University of Medical Sciences, Tabriz, Iran; *; c * Dental and Periodontal Research Center, Department of Endodontics, Dental School, Tabriz University of Medical Sciences, Tabriz, Iran; *; d *Department of Endodontics, Dental School, Tabriz University of Medical Sciences, Tabriz, Iran*

**Keywords:** Manual Instrumentation, Postoperative Pain, Root Canal Preparation, Rotary Instrumentation

## Abstract

**Introduction:**

One of the most common complications of root canal treatment is postoperative pain. The aim of the present clinical trial was to compare the severity of postoperative pain after root canal preparation with RaCe rotary system and hand K-Flexofile.

**Methods and Materials:**

A total of 96 mandibular first and second molars were divided into two groups (*n*=48) based on root canal preparation technique. The teeth in both groups underwent one-session root canal treatment and the severity of postoperative pain was evaluated using visual analog scale (VAS) at 4-, 8-, 12-, 24- and 48-h and 1-week intervals. In addition, the type and dosage of analgesics were recorded. Data were analyzed with repeated-measures ANOVA. Statistical significance was set at 0.05.

**Results:**

The difference between the two groups during this period and at subsequent intervals were not significant (*P*>0.05). There were no significant differences between the two groups in type and the number of analgesics in pain-free subjects (*P*=0.12 and *P*=0.61, respectively).

**Conclusion:**

There were no statistically significant differences in pain severity between the two groups at any intervals.

## Introduction

Postoperative pain is a common complication in endodontic treatment with the occurrence of 1.4-16% [1-3]. Age, gender, tooth type, pulp status, presence of sinus tracts and sensitivity and preoperative pain have been reported as risk factors that may affect the incidence of postoperative pain after root canal therapy [2]. 

There is some supportive evidence that one of the most important reasons for postoperative endodontic pain is the extrusion of infected debris from the root apex during chemomechanical debridement, which results in an acute inflammatory response [4]. There are various claims about the ability of some rotary techniques to minimize the extrusion of debris in comparison to other techniques [2]. Rotary instruments result in the extrusion of less debris compared to stainless steel hand files due to their rotational movements (Archimedes screw effect), leading to less postoperative and discomfort when they are combined with copious irrigation [2, 5]. 

One of the most commonly used rotary systems is RaCe (FKG Dentaire, La-Chaux-de Fonds, Switzerland) system, which is mainly used with the crown-down technique. The design includes two grooves followed by one straight area without any grooves along the file, which appears to be an area for accumulation and evacuation of debris and can result in a decrease in screw-in effect in association with the use of the crown-down technique and enlargement of the coronal area of the root canal. This design increases the evacuation of debris from the coronal area of the root canal and decreases the extrusion of debris from the root apex, which might result in less severe postoperative pain [6-8]. Forogh Reihani *et al.* [9] showed significantly less extrusion of debris with the use of RaCe instruments compared to Mtwo system.

Several studies have shown that there is no clear-cut and significant relationship between extrusion of debris *in vitro* and postoperative pain under clinical circumstances. In this context, despite an increase in the extrusion of debris with the use of hand files compared to rotary files in various studies, in some studies no significant relationship has been found between them regarding the postoperative pain, which might indicate the role of other factors, in addition to the extrusion of debris, in the severity of postoperative pain [10-12].

Based on the results of previous studies [10-14], it appears that the use of rotary systems does not guarantee a decrease in postoperative endodontic pain. Furthermore, there are discrepancies in the results of previous studies and there is only limited number of randomized clinical trials about the postoperative pain after the application of rotary and hand files and no study is available to compare postoperative pain after the use of RaCe rotary files and K-Flexofile. Therefore, the present prospective randomized controlled clinical trial was undertaken to compare the intensity of postoperative endodontic pain subsequent to endodontic treatment using either RaCe rotary instruments or hand K-Flexofiles.

## Materials and Methods

This prospective randomized controlled clinical trial was approved by the Ethics Committee of Tabriz University of Medical Sciences (Grant No.: 93190) and registered at the Iranian Registry of Clinical Trials (Registration ID: IRCT2015041521780N1). The present study was carried out on patients referring to the Department of Endodontics, Tabriz Faculty of Dentistry from September 2014 to April 2015. The patients were unaware of the technique used for endodontic treatment, and visual analog scale (VAS) data analysis was carried out by a blinded operator. Therefore, the study was carried out in a double-blind manner.

**Table 1:** Inclusion and exclusion criteria for patient retrieval

	**Inclusion**	**Exclusion**
**Age**	Over 18	Under 18
**Pulp status**	Asymptomatic irreversible pulpitis	Healthy Reversible pulpitis Symptomatic irreversible pulpitis Necrosis
**Periradicular status**	Normal radiographically	Widening of PDL Radiolucency Radiopacity
**General health status**	Healthy	Systemic disease Breast-feeding Pregnancy Hypersensitivity to lidocaine
**Restorative status**	Conducive to restoration	Impossible to restore Presence of a crown before the procedure
**Treatment plan**	Needing selective one-visit treatment	Needing 2-visit treatment or any extra procedure such as incision and drainage or more teeth needing RCT on the same side
**During treatment**	-Presence of lip sign after administration of anesthesia -Presence of bleeding after exposure of the pulp	Absence of lip sign after administration of anesthesia Absence of bleeding after exposure of the pulp over instrumentation or over filling
**Number of root canals**	3 or 4 root canals	1 or 2 root canals The presence of a difficult root canal anatomy (root canals with extreme curvatures (over 30°) and C-shape canals) Internal or external resorption Teeth with open apices Radiographically untraceable canal path or any accident or complication occurring during treatment (calcified canals, inability to achieve apical patency in any canal)
**Drug therapy**	No drug use or use of 400 mg of ibuprofen during the 24-h postoperative interval	Use of analgesics 12 h before treatment, Use of more than 400 mg of ibuprofen during the 24-h postoperative interval or any dose after 24 h
**Tooth position**	Straight, not difficult to access SLA	Severe labial or lingual malpositioning making it difficult to access SLA

The sample size was estimated based on the results of a pilot study. The final sample size was calculated to be 96 subjects, with 48 mandibular molars in each group by considering the results of the plot study and considering *α*=0.05 and a study power of 80%. 

The inclusion criteria consisted of otherwise healthy subjects requiring endodontic treatment on mandibular first or second molars with asymptomatic irreversible pulpits with normal periapical radiographic views as shown in Table 1. The clinical diagnosis of asymptomatic irreversible pulpitis was based on increased response to cold test with Green Endo-ice (1,1,1,2-tetrafluoroethane; Hygenic Corp, Acron, OH, USA) and the presence of deep caries on the radiographies, extending to the pulp space, without any symptoms. Patients with sinus tracts, periapical abscesses and the patients with other problems listed in Table 1 were excluded from the study.

Pulp vitality and periradicular status of each tooth was evaluated with thermal and electric pulp tests (Diagnostic Unit; Sybron, Orange, CA, USA), followed by palpation and percussion and periodontal charting. Periapical radiographies (Intra, Planmeca, Helsinki, Finland) were used for further examinations with Rinn XCP devices (Rinn Corp, Elgin, IL, USA) and a digital radiographic system (RVG 5100; Eastman Kodak Co, Rochester, NY, USA) and processed and archived by a special scanner and software interface (Optime, Soredex, Finland). Clinical and radiologic data were analyzed by three independent and blinded operators.

After selecting 96 eligible subjects, a clinician blinded to the treatment in each group randomly divided them into two equal groups of 48 subjects each. The two groups were matched regarding gender and the number of mandibular first and second molars with 3 and 4 root canals. 

Before initiating the study procedures, the advantages and outcomes of the procedures were thoroughly explained to the subjects and informed written consent was obtained from each subject in both groups. The patients were categorized in treatment groups by selecting a pocket that indicated the method of instrumentation. 

***Endodontic procedures***

Inferior alveolar nerve block anesthesia was administered with injection of 1.8 mL of lidocaine containing 1:80000 epinephrine (Daroupakhsh, Tehran, Iran), followed by buccal infiltration of long buccal nerve for easier replacement of rubber dam. After numbness of the lip, cold and electric tests were used to confirm pulpal anesthesia. In cases with no local anesthesia, supplementary injections (intraligamentary injection) were used, which was recorded in patient forms. After confirmation of anesthesia, access cavity preparation, observation of pulpal hemorrhage and confirmation of pulp vitality, the tooth was isolated with rubber dam. The root canal orifices were located. In group one, RaCe rotary instruments were used to prepare the root canals using modified crown-down technique according to the manufacturer’s instructions. The working length (WL) was estimated with the use of a preoperative radiographies, using #10 hand K-file (Dentsply Maillefer, Ballaigues, Switzerland). In order to prepare the coronal and middle thirds of the root canals, 40/0.10, 35/0.08 and 30/0.06 files were used. The WL was meticulously determined with the use of Root ZX apex locator (Morita Corp., Tokyo, Japan) and a #10 hand K-file, which was confirmed with the use of a digital radiographies.

The apical third of the root canals was prepared with a 25/0.02 files up to the WL. If resistance was encountered, #15 and #20 hand files were used, and preparation was followed by the use of 25/0.04 and 25/0.06 instruments. In cases where the #20 hand file did not reach the WL, the sample was excluded from the study. Apical preparation of the mesial and distal root canals of molars with 4 root canals and the mesial root canals of molars with 3 root canals continued up to 30/0.04 files. In molars with 3 root canals, apical preparation of the distal root canal continued up to 35/0.04 file. 

In the manual root canal preparation group, root canal preparation continued with stainless steel hand K-files and K-Flexofiles using the step-back technique. At this stage, the presence of a glide path at the coronal zone of the root canals were evaluated at the estimated WL with the use of a preoperative radiography using a #10 hand K-file. The WL was determined with a Root ZX apex locator, using a #10 hand K-file and confirmed by a digital radiographic system. Preparation of the apical third began with the use of the largest file, considered as the initial file, which reached the full WL determined with the use of the electronic apex locater and radiography. Preparation continued up to three sizes larger than the initial file, which was considered as the master apical file (MAF). Hand K-files #10 and 15 and #20, 25 and 30 Flexofiles were used to prepare the apical thirds of mesial and distal root canals of molars with 4 root canals and files #35 were used to prepare the apical thirds of distal root canals of three-rooted molars. The middle and coronal thirds of the root canals were prepared with consecutive increases in file sizes and by a 0.5-mm decrease in WL in each larger file up to 4 sizes, based on the coronal width. Files larger than the MAF were used to prepare the middle and coronal thirds with the use of feed-it-and-pull movements based on Ruddles technique, in which hand files are used with -1/4 and +1/4 apical movements up to a point at which resistance is encountered, followed by slow backward pulling of the files so that the debris is removed from the root canal. This procedure continued to reach the target root canal length with each file [15]. During all the preparation procedures with both rotary and manual techniques, 10 mL of 5% NaOCl was used for irrigation of the root canals with a 30-G needle syringe.

**Figure 1:**
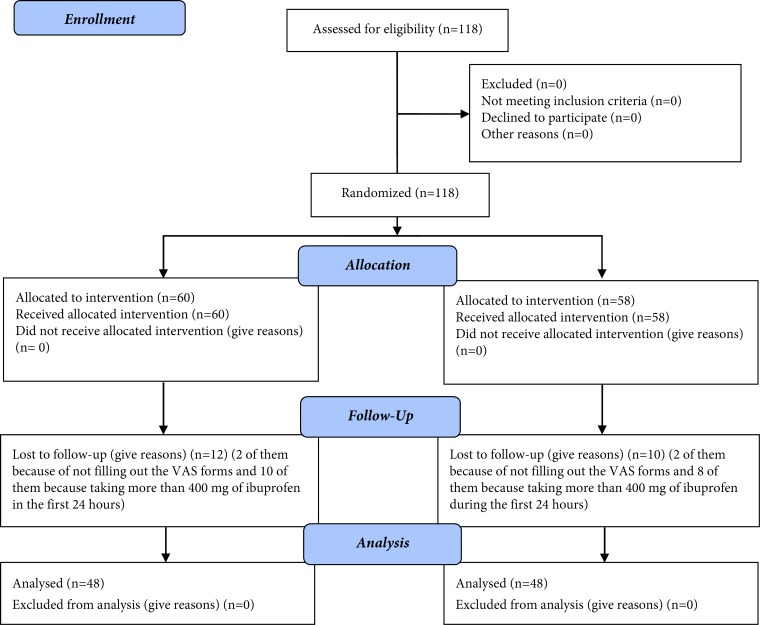
CONSORT 2010 Flow chart

After preparation procedures, the root canals were dried with paper points and obturated during the same session using lateral compaction of gutta-percha (Meta Biomed Co., Cheongju City, Chungbuk, Korea) and AH-Plus sealer (Dentsply DeTrey, Konstanz, Germany). Finally, the access cavity in each tooth was sealed with eugenol temporary dressing (Zonalin; Associated Dental Products, Wiltshire, United Kingdom) and the patient’s occlusion was checked to make sure that the temporary dressing did not interfere with occlusion. 

Postoperative pain was evaluated with VAS. VAS consisted of a straight line graduated from 1 to 100 and is used to evaluate pain severity from “no pain” to the “most severe pain conceivable”. The severity of pain is marked on the line by patients at each of the time intervals [16]. The picture was given to each patient and the filling technique was explained orally and in written form. A total of 6 VAS pictures were handed in to the patients, *i.e.* one picture for each time interval.

Data on pain was recorded by the patients at 4-, 8-, 12-, 24- and 48-h and 1-week intervals. The patients were instructed to take mild analgesics (400 mg of ibuprofen) (Gelofen; Jaber Ebne Hayyan Pharmaceutical Mfg. Co., Tehran, Iran) in case of pain. Patients taking 400 mg of ibuprofen during the first 24 h in each group were considered to have moderate pain (a mean score of 30 on the VAS according to Table 2) at 4-, 8- and 12-h intervals [3].

Since the dose-dependent activity of ibuprofen is 4 to 8 h, which is longer than its half-life (~2 h) and since its analgesic effect completely disappears in 8 h [17], these patients were evaluated at 24- and 48-h and 1-week intervals similar to other patients in the study. Patients taking more than 400 mg of ibuprofen during the first 24 h and those taking any dose of the medicine after 24 h were excluded from the study. 

Data were analyzed with repeated-measures ANOVA, using SPSS software (Statistical Package for Social Science, SPSS, version 17.0, SPSS, Chicago, IL, USA). Post hoc tests were used for two-by-two comparisons. Statistical significance was set at 0.05.

## Results

A total of 118 patients contributed to this study. Four patients were missed because of not filling out the VAS forms and 18 were excluded based on the exclusion criteria of the study (Figure 1). In both manual and rotary groups, severity of postoperative pain significantly decreased from the beginning to the end at all evaluated time intervals (*P*<0.001). 

However, comparison of pain severity between the RaCe rotary and hand K-Flexofile groups did not reveal any significant differences between the two groups (*P*=0.84). In this context, the mean pain severity scores 4 h after treatment were 26.91±4.20 in the RaCe group and 34.39±4.62 in the K-Flexofile group. After 8 h, the pain severity scores were 20.14±3.94 and 23.31±3.89 in the rotary and hand file groups, respectively. The pain severity at both intervals in the rotary group was less than the hand file group, but the difference was not statistically significant (*P*>0.05). At 12-, 24- and 48-h and 1-week intervals, either, the differences in pain severity between the two groups were not significant (*P*>0.05) (Table 3).

Despite more severe postoperative pain during the first 8 h in the hand file group, the rate of decrease in pain severity was higher in this group compared to the rotary file group. On the other hand, during the first 4 h, 11 subjects (22.9%) in the rotary file group and 9 subjects (18.8%) in the hand file group had no pain, with no significant differences between the two groups in the number of pain-free subjects at any time interval (*P*=0.61). 

The number of patients taking analgesics during the first 24-h postoperative period was 22 (45.8%) and 15 (31.3%) in the hand and rotary file groups, respectively, with no significant differences between the two groups (*P*=0.12).

**Table 2:** Pain severities at different time intervals

**Severity**	**Distance (X)**
**No pain**	x=0 mm
**Mild**	20 mm≥x>0 mm
**Moderate**	40 mm≥x>20 mm
**Severe**	60 mm≥x>40 mm
**Very severe**	80 mm≥x>60 mm
**The most severe pain conceivable**	x>80 mm

## Discussion

The aim of the present prospective randomized controlled clinical trial was to compare the effect of root canal treatment with hand K-Flexofiles and rotary RaCe files on the incidence and intensity of postoperative endodontic pain. 

Based on the results of the present study, comparison of pain severity between the two groups at different time intervals showed no significant differences. However, more severe pain was detected in the hand file group during the first 8 h after completion of the treatment and more severe pain in the rotary file group at 12-, 24- and 48-h and 1-week postoperative intervals. In addition, there were no significant differences between the two groups regarding the number of patients taking analgesics during the first 24-h postoperative interval. 

The subjective nature of postoperative pain is a source of difficulty in such studies, which depends on the cultural, individual and economic background of the subjects. Evaluation of pain is inherently difficult; therefore, in the present study the subjects received adequate explanations about postoperative pain and VAS. Most subjects understand VAS technique easily and are able to rate their pain severity. VAS is considered a reliable and valid technique for evaluation of pain relief [2, 18]. In this context, the two groups of the study were matched in relation to age, gender, tooth type, and pulp and periapical status. In addition, all the technique- and operator-related variables were controlled since one single operator performed all the root canal therapy (RCT) procedures; the only differences were the file type and instrumentation technique in two separate groups.

A recent systematic review reported an incidence rate of 40% for postoperative pain during a 24-h period, which decreased significantly during the first 48 h after treatment, with 10% or less after 7 days [19], and is consistent with the present study. Studies have shown that one of the most important reasons for postoperative pain is the extrusion of debris from the root end during chemomechanical debridement, resulting in an acute inflammatory response [4, 20]. Different factors affect the extrusion of debris, including the technique used for irrigation, the volume and concentration of irrigation solution, the final apical size, the anatomical features of the apical constriction, all of which were observed in the present study [21-30].

**Table 3:** Pain severities in two groups based on VAS

	**Rotary**	**Manual **
**4 h**	26.91±4.20	34.39±4.62
**8 h**	20.14±3.94	23.31±3.89
**12 h**	17.20±3.65	12.37±2.86
**24 h**	14.66±3.65	8.91±2.468
**48 h**	9.64±2.63	5.18±1.93
**1 w**	2.87±1.18	2.70±1.69

In this study root canal treatments were completed in one visit to minimize the effect of related variables and the treated teeth in both groups were relieved of any premature occlusal contacts after treatment so that inappropriate occlusal contacts or trauma from occlusion would not affect the results.

One of the problems in evaluating postoperative pain is the possible role of pain mediators such as substance P (SP) and calcitonin gene-related peptide (CGRP). In this context, Caviedes-Bucheli *et al.* [31] evaluated the expression of SP and CGRP in the periodontal ligament of human after the use of single-file reciprocating systems and reported that more neuropeptides were expressed in teeth undergoing endodontic treatment with the Wave One system, concluding that the design of the files minimize the coronal transportation of dentinal debris and increased apical extrusion of debris, which gave rise to a higher neuropeptide concentration. It should be noted that SP and CGRP activate G protein-coupled receptors on nociceptors and thus sensitize or activate neurons [32]. In addition these neuropeptides result in peripheral sensitization manifested as hyperalgesia, allodynia and spontaneous pain [33]. Moreover, an increase in barrage in inputs with sufficient intensity and duration leads to central sensitization, suggesting that both peripheral and central sensitization may play a role in pain experience in patients with more extruded debris [1].

In the present study, the pulp and periapical status of the teeth were matched in both groups to prevent the possible effect of inflammatory mediators on postoperative pain with the use of rotary system and hand files.

Researchers attributed differences in the extrusion of debris from the root apex to differences in root canal preparation techniques, cervical preflaring, type of tooth and instrument designs [34-36]. The design of RaCe system used in the present study has been shown in extracted teeth to lead to less extrusion of debris from the apical area, which might decrease postoperative pain severity [7]. In this context, due to the advantages mentioned for this system, postoperative pain during the first 8 h after completion of the treatment in the RaCe rotary group was less than the hand file group, with fewer analgesics taken in the RaCe file group during the first 24 h.

In an *in vitro* study by Yeter *et al. *[12], there were no significant differences in extrusion of debris between Revo- system rotary files and hand K-files. In another *in vitro* study, Vaudt *et al.* [5], compared root canal preparation with two NiTi rotary systems (Alpha and Protaper Universal Systems) and stainless steel hand files. Less debris was extruded with the use of the two rotary systems compared to hand K-files [5]. Similar to the present study, both aforementioned assessments used the crown-down technique to prepare the root canals in the rotary system groups and it was reported that use of the crown-down technique can decrease extrusion of debris from the root apex and the subsequent postoperative pain severity by enlarging the coronal third of the root canal and providing a path for the exit of debris from the root canals [5, 6]. In addition, in studies mentioned above, the step-back technique and pull-and-push movements were used for root canal preparation in the hand file groups and as it has been shown in previous studies, large amounts of debris and irrigation solutions are extruded with the use of the step-back technique, which has been attributed to the watch-winding and in-and-out filing motions, resulting in piston-like movements and extrusion of more debris and irrigation solutions compared to other instrumentation techniques. In the crown-down technique, the coronal area is prepared first and then the apical area is prepared, which results in the extrusion of less debris [35]. However, in the present study, despite the use of the step-back technique, feed-it-and-pull movements were used based on Ruddle’s technique instead of pull-and-push movements for flaring of the root canals. This type of file movement does not lead to extrusion of debris from the root canal. This technique appears to help debris undergo suspension in the irrigation solution, minimizing the odds of postoperative pain [15] in manual group similar to RaCe rotary group in the present study. It should be pointed out that the clinical results might be different because periapical tissues serve as a natural barrier against extrusion of debris, preventing extending the results to clinical situations.

Another important consideration in the present study was comparison of the time required to prepare the root canals in the rotary and hand file groups. Despite the use of more numerous rotary files compared to the routine procedures in the rotary group, root canal preparation with hand K-Flexofiles took longer compared to that with RaCe rotary system; therefore, there was longer contact with root canal walls, resulting in the production of more debris and more manipulation of the apical area and increasing the postoperative pain in hand file group during the first 8 h.

## Conclusion

Considering the lack of significant differences in the severity of postoperative pain between the RaCe rotary and hand K-Flexofiles, it appears use of the crown-down technique is more effective in postoperative pain than the file type. Therefore, it is suggested that future studies evaluate the hand and rotary files with the same crown-down technique in both groups.
